# Experimental dataset on tailoring hematite nanodots embedded nitrogen-rich carbon layers for lithium-ion batteries

**DOI:** 10.1016/j.dib.2020.105472

**Published:** 2020-04-05

**Authors:** Chenrayan Senthil, Chang Woo Lee

**Affiliations:** Department of Chemical Engineering & Center for the SMART Energy Platform, College of Engineering, Kyung Hee University, 1732 Deogyeong-daero, Giheung, Yongin, Gyeonggi, 17104. South Korea

**Keywords:** Fe_2_O_3_ nanodot, Morphology, Anode, Lithium-ion battery

## Abstract

The experimental data presented are related to the research article entitled “Nitrogen self-doped carbon sheets anchored hematite nanodots as efficient Li-ion storage anodes through pseudocapacitance mediated redox process” [1]. In brief, the synthesis of nanodotted hematite Fe_2_O_3_ embedded in nitrogen-rich carbon layers is achieved through a surfactant-less self-assembly process and it is employed as anodes for Li-ion batteries. The dataset presented depicts the effect of temperature on the phase formation and morphology of the Fe_2_O_3_ nanodots and their influence on the electrochemical performance by constructing as anode materials for lithium-ion batteries. Representative XRD patterns, FE-SEM and FE-TEM micrographs, electrochemical potential profiles, and cycling performances for anode materials synthesized by different thermal treatment process are investigated. The shared datasets contribute to clarify the formation temperature and morphological evolution of Fe_2_O_3_ into nanodots.

Specifications table

**Table:** 

Subject	Chemistry
Specific subject area	Inorganic Chemistry and Electrochemistry
Type of data	Figures
How data were acquired	XRD (Panalytical X'pert Pro, PHILIPS), FE-SEM (JSM, JEOL), FE-TEM (JEM2100F, JEOL), EDS Mapping (Oxford Instrument), Electrochemical Cycling (BioLogic VSP300, ETH Instrument), Battery test-vector (CR2032 coin-type cells).
Data format	Raw, Analysed
Parameters for data collection	Powder samples for physico-chemical characterization electrochemical characterization: Profile and capacity retention data measurements (potential sweep between 0.005 to 3.0 V at 0.1 *C* ≈ 100 mA). All the electrochemical measurements were carried out in room temperature (≈25 °C).
Description of data collection	Electrochemical data was collected after fabricating the coin-cells with an equilibration time of 2 h. The raw dataset containing the electrochemical studies are provided as supplementary material which are measured between the voltage window of 0.005 to 3.0 V at a current rate of 0.1 C against Li/Li^+^in room temperature (≈25 °C).
Data source location	SRM IST, Chennai, India and KHU, Yongin, South Korea
Data accessibility	Data provided in this article and supplementary data for battery studies
Related research article	*S. Chenrayan, A. Subramani, P. Thamodaran, N. Mani, K. Vediappan,* S. Manickam,* C.W. Lee* Nitrogen self-doped carbon sheets anchored hematite nanodots as efficient Li-ion storage anodes through pseudocapacitance mediated redox process J. Ind. Eng. Chem. 85 (2020) 289–296. https://doi.org/10.1016/j.jiec.2020.02.014.

## Value of the data

• Significant understanding on the effect of temperature towards the phase and morphological evolution to Fe_2_O_3_ nanodots.
• Data on temperature dependant formation of nitrogen-doped carbon layers from carbon nitrides.
• Data provides understanding on the structure and electrochemical reactivity with respect to temperature.

## 1 Data

**Fig. 1:**
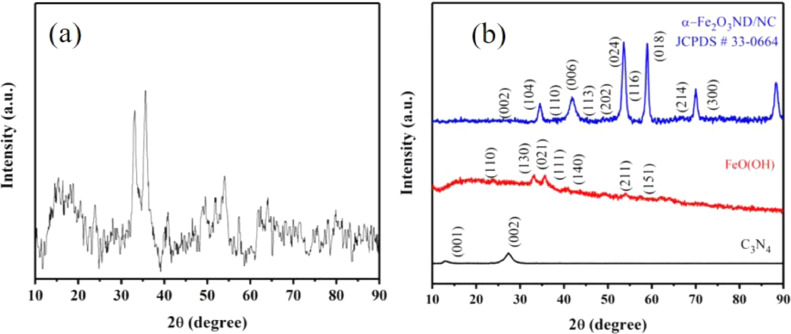
XRD patterns of samples prepared at (a) 350 °C and (b) 450 °C.

**Fig. 2:**
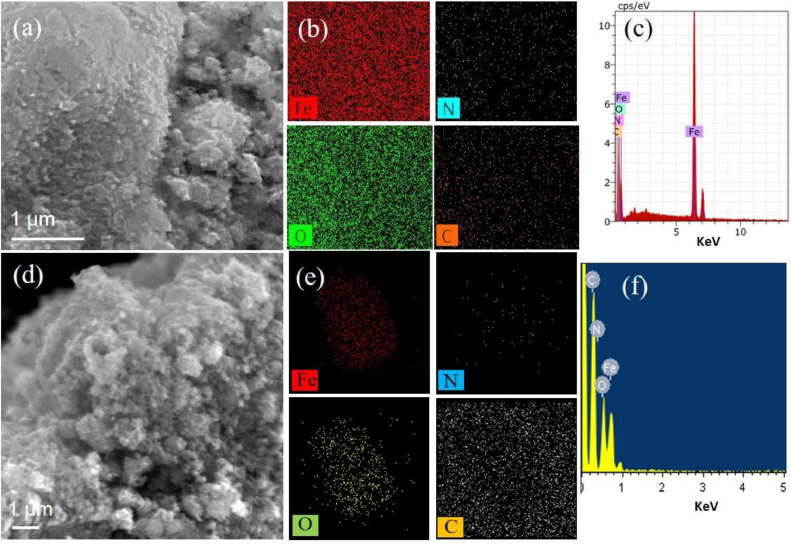
(a and d) FE-SEM micrographs, (b and e) EDS mapping, and (c and f) EDAX spectrum for Fe_2_O_3_ prepared at 350 °C and 450 °C, respectively.

**Fig. 3:**
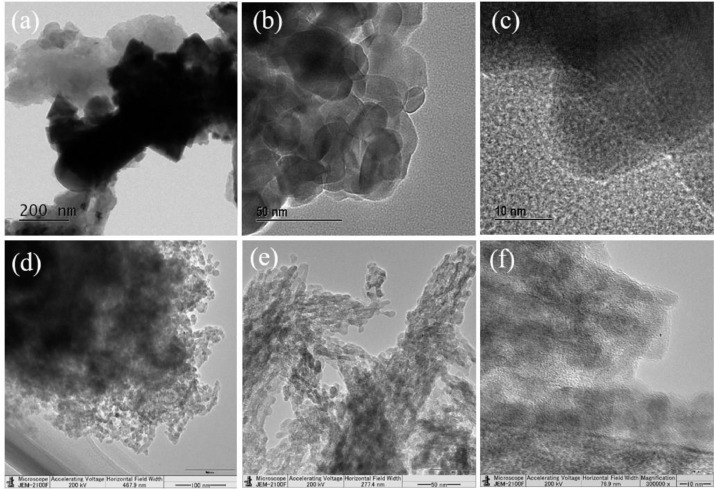
FE-TEM micrographs of (a) C_3_N_4_ and (b&c) and (d-f) Fe_2_O_3_ /nitrogen-rich carbon layers prepared at 350 °C and 450 °C, respectively.

**Fig. 4:**
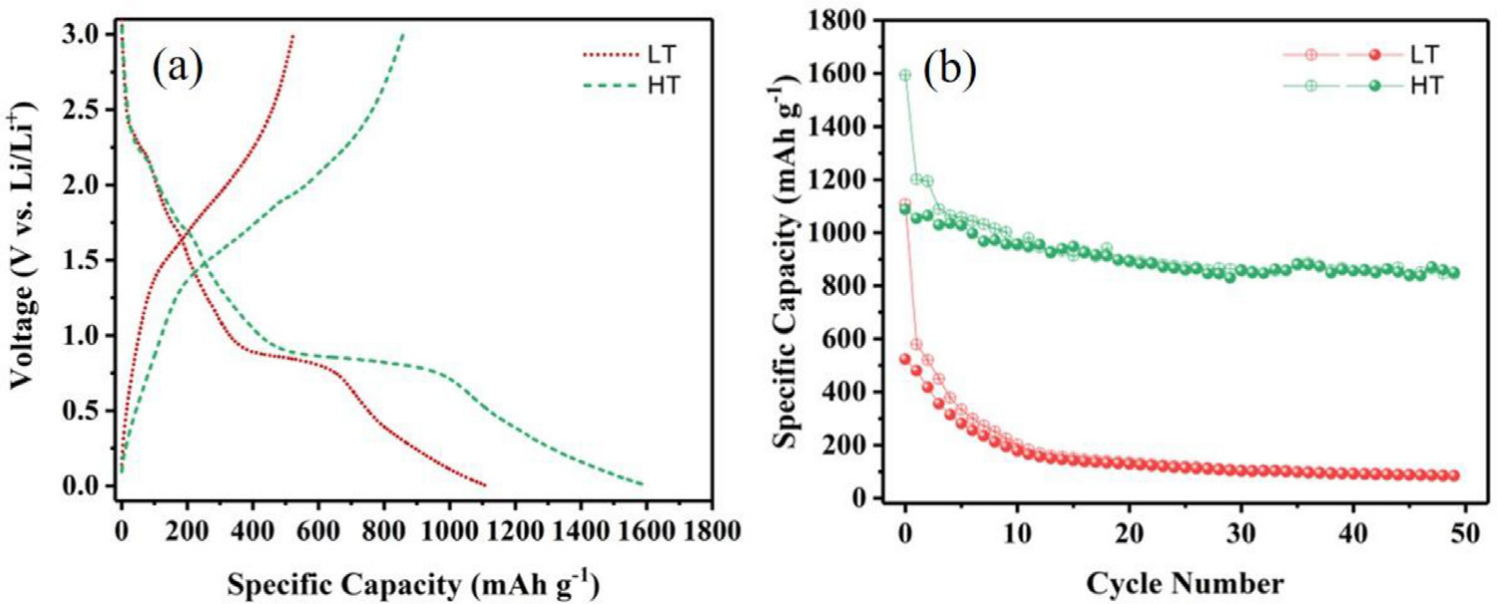
Electrochemical performances of the materials prepared at 350 °C (LT) and 450 °C (HT); (a) potential profile and (b) capacity retention.

The experimental data presented in this dataset have been generated on tailoring the hematite Fe_2_O_3_ into nanodots embedded in nitrogen-rich carbon layers through a surfactant-less and self-assembly approach. Fig. 1 shows the XRD patterns of as-prepared materials with respect to the effect of thermal treatment process. Figs. 2 and 3 show the micrographs obtained from FE-SEM and FE-TEM analyses depicting the morphology and elemental occurrence of elements with respect to different temperatures and their corresponding electrochemical performances are shown in Fig. 4.

## 2 Experimental design, materials, and methods

Detailed experimental procedure for the preparation of carbon nitrides and their exfoliation to carbon nitride nanosheets and Fe_2_O_3_ nanodots embedded in nitrogen-rich carbon layers are provided in reference [1]. In brief, nanosheets of carbon nitrides were prepared through the chemical exfoliation of bulk carbon nitrides, which were used as self-template to confine Fe_2_O_3_ into nanodots embedded in nitrogen-doped carbon layers. In order to investigate the proper heat-treatment temperature to attain hematite (α-Fe_2_O_3_) phase, materials were thermally treated at different temperature. The material prepared at 350 °C reveals a semi-crystalline diffraction pattern as shown in Fig. 1a. However, the material prepared at 450 °C exposes clear diffraction patterns that could be indexed to the hematite phase as shown in Fig. 1b.

The morphology, elemental mapping and distribution of the elements for the as-prepared materials at different temperatures, 350 and 450 °C are shown in Fig. 2a–f, respectively. In both the cases, the as-prepared material showed agglomerated clusters consisting of tiny sized particles. Fig. 2b and c and Fig. 2e and f show the respective elemental mapping and EDAX spectrum of sample obtained at 350 °C exhibiting the occurrence of Fe, O, C, and N in the sample. In addition, the mapping data expose the uniform distribution of nitrogen in the carbon which embeds the Fe_2_O_3_ particles.

In general, the carbon as conductive network is introduced in the second step, i.e., post-preparation of the material. FE-TEM micrograph shown in Fig. 3a represents carbon nitride depicting the thin sheet like structure, which is used as self-template to fix the metal particles and in-situ transformation of nitrogen-doped carbon layers. Fig. 3b–c and d–f show the micrographs of samples prepared at 350 °C and 450 °C, respectively, whereas the later one shows confined formation of Fe_2_O_3_ particles on nitrogen-doped carbon layers.

The derived profile plots for the Fe_2_O_3_ samples prepared at different temperatures are shown in Fig. 4a. The initial discharge and charge capacities of the samples prepared at 350 °C and 450 °C are 1109 and 538 mAh g^−1^ and 1594 and 1086 mAh g^−1^, respectively, with a coulombic efficiency of 48.5% and 68.1%. The represented specific capacity values (dataset provided as supplementary material) are solely based on the active material, i.e., Fe_2_O_3_ content in the electrodes.

Fig. 4b depicts the capacity retention for the 350 °C and 450 °C materials (raw dataset provided as supplementary material), where the later one delivered a higher reversible capacity of 926 mAh g^−1^ at the end of 50 cycles compared to former material with 93 mAh g^−1^.

## Appendix Supplementary materials

Image, application 1
